# A Century of General Lichtenecker Equation: Between Stringency and Empiricism, Accuracy and Approximability

**DOI:** 10.3390/ma18245562

**Published:** 2025-12-11

**Authors:** Anatoliy V. Goncharenko, Vyacheslav M. Silkin

**Affiliations:** 1 V.E. Lashkaryov Institute of Semiconductor Physics, 41 Nauky Ave., 03028 Kyiv, Ukraine; 2 Donostia International Physics Center (DIPC), 20018 San Sebastian, Basque Country, Spain; 3 Departamento de Polímeros y Materiales Avanzados: Física, Química y Tecnología, Facultad de Ciencias Químicas, Universidad del País Vasco UPV/EHU, 20080 San Sebastian, Basque Country, Spain; 4 IKERBASQUE, Basque Foundation for Science, 48013 Bilbao, Basque Country, Spain

**Keywords:** effective medium, homogenization, Lichtenecker equation, Archie’s law, spectral representation

## Abstract

The general Lichtenecker equation, with exponent values in the range −1≤k≤1, is widely used to describe and predict the effective macroscopic electrical, magnetic, thermal and optical properties of various heterogeneous media. Although it sometimes fits experimental data well, in most cases, however, it is physically incorrect and its predictive ability is often overestimated. Using a rigorous spectral representation, we show that for isotropic composites, except for the trivial one-dimensional case, only two specific forms of this equation, the logarithmic (k=0) and Landau-Lifshitz-Looyenga (k=1/3) mixing laws, are dimensionally consistent, corresponding to spatial dimensions d=2 and d=3, respectively. Furthermore, the requirement of phase-interchange symmetry severely restricts the applicability of the Lichtenecker equation, rendering it unsuitable for most real composite systems.

## 1. Introduction

Many researchers and engineers face the problem of expressing the effective macroscopic response (such as permittivity, conductivity, diffusivity, etc.) of various heterogeneous media, including dielectric composites, porous materials, disordered systems. Perhaps the most popular approach is based on phenomenological effective medium (homogenization) theories. Three of them, associated with the names of Maxwell Garnett, Bruggeman and Lichtenecker, are especially frequently used.

In 2026, it is a century as Karl Lichtenecker first put forward his empirical mixing rules [1]. The original equation for the effective permittivity $\epsilon_{eff}$ in its general form was

(1) $$\epsilon_{eff}^{k}=f_{1}\epsilon_{1}^{k}+f_{2}\epsilon_{2}^{k},$$

where $f_{1}$ and $f_{2}$ are the volume fractions of the phases 1 and 2, respectively, $\epsilon_{1}$ and $\epsilon_{2}$ are their permittivities, and *k* is a phenomenoligical parameter. The value of *k* in Equation (1) is constrained by the Wiener bounds, i.e., −1≤k≤1, and is often considered as a fitting parameter.

In his earlier paper [2], Lichtenecker also proposed an equation of the form

(2) $$\log\left(\epsilon_{eff}\right)=f_{1}\log\left(\epsilon_{1}\right)+f_{2}\log\left(\epsilon_{2}\right),$$

which can in fact be considered as a particular case of Equation (1) in the limit k→0. The main assumption made in deriving Equation (2) is a random distribution of particle shapes and orientations for each phase such that the charge density within any phase can be replaced by the mean charge density of the mixture. This allows one to replace the charge fractions with their volume fractions [3].

In the literature, Equation (2) is also frequently called as Lichtenecker equation. To avoid confusion, in the following we refer to Equation (1) with arbitrary *k* as the general Lichtenecker equation (LE), while Equation (2) is referred to as the logarithmic Lichtenecker equation (LLE). As can be seen, Equation (1) represents the weighted arithmetic and harmonic means at k=1 and k=−1, respectively, while Equation (2) represents the weighted geometric mean.

The LE has been widely used or tested to describe and predict effective permittivity, permeability, electric, thermal and hydraulic conductivity of diverse natural and artificial mixtures—liquids, solids, powders, metal composites, etc. Particular examples include the permittivity of emulsions [4,5], soils [6], multiwalled carbon nanotube/polymer nanocomposites [7], nanoparticle doped elastomeric polymers [8], fabric aggregates [9], metal nanopowders [10,11], concrete [12], Lunar and asteroid regolith [13], barium titanate based composites [14], alcali brick composites [15], piezoelectric ceramics [16]; the electric conductivity of shaly sandstones [17] and sedimentary rocks [18]; the thermal conductivity of mineral wools [19] and sedimentary rocks [20]; the magnetic permeability of ferrite/polymer composites [21,22].

While the values of the parameter *k* may lie within the range from −1 to 1, both edges of this interval are associated with a strong anisotropy [16,23,24]. In this study we focus mainly on isotropic systems. Our aim is to clarify the physical premises underlying the LE and to establish a set of fundamental limitations that govern its validity. While this equation is widely used, it appears to be overstated and, in fact, its area of applicability is rather limited. In the following we show this using simple physical arguments involving the formalism of spectral representation.

## 2. Geometry and Percolation

Taking look at Equation (1), several immediate conclusions can be made. First, it is a symmetric approximation in which both phases are treated equally. This means that the matrix (host) and inclusions are indistinguishable: every phase can be regarded as the host or as the inclusion, and the corresponding composite media should possess phase-inversion symmetry [25]. Thus, the microgeometry of each phase is completely identical to that of the other. The geometry must be such that both phases interpenetrate in a comparable manner (neither is clearly an inclusion within the other), as in bicontinuous or lamellar structures, symmetric random blends, etc. If we swap phases 1 and 2, the geometry remains the same and the effective property relation remains unchanged, that reflects its invariance.

Another important point is that, as the LE assumes, for the corresponding microgeometry both phases must topologically percolate at any volume fractions $f_{i}$, if only k≥0. Indeed, as is easy to see that, in terms of conductivity, $\sigma_{eff}\to \infty$ if any $\sigma_{i}\to \infty$.

Most real composite systems, of course, do not satisfy the above conditions of symmetry and percolation. Moreover, as will be shown below, even if both above conditions are met, it does not necessarily follow that Equation (1) holds.

## 3. Spectral Density Function Analysis

To take a step forward, let us now consider the LE in terms of the spectral representation. It should be noted that this representation is conceptually analogous to the rigorous Green´s function formalism, which is known to be the backbone of many approaches in condensed matter physics. Within the framework of the spectral representation, the effective permittivity $\tilde{\epsilon }$ can be expressed as [26,27,28,29]

(3) $${\tilde{\epsilon }}_{eff}/\epsilon_{1}\equiv \tilde{\epsilon }=1+f_{2}\left(\frac{C_{2}}{s_{2}}+\int_{0}^{1}\frac{g_{2}\left(x\right)dx}{s_{2}+x}\right),$$

where $s_{2}={\left(\epsilon_{2}/\epsilon_{1}-1\right)}^{-1}$ and *x* plays the role of a spectral variable linked to depolarization factors. The function $g_{2}\left(x\right)$ contains the necessary information about the microgeometry of the composite and is referred to as the spectral density function (SDF) [28,30,31,32]. The SDF is similar to the density of states (DOS) in electronic structure theory which, in turn, is known to have a profound significance in disordered systems, where the Bloch theory becomes inapplicable. While the DOS gives us the distribution of electronic states over energy, independent of the specific interaction strength, the SDF specifies how geometrical modes, associated with polarization resonances, are distributed, independent of the material constants of constituents. Thus, the SDF highlights how disorder and geometry manifest themselves as distributions of modes, rather than through deterministic parameters.

Any effective material property, such as permittivity, permeability, electric, thermal or hydraulic conductivity, or a diffusion coefficient can be expressed as a function of a contrast parameter *h*, which can be defined as $h=\epsilon_{2}/\epsilon_{1}$ or $h=\sigma_{2}/\sigma_{1}$ in terms of the permittivity or electric conductivity, respectively. For electromagnetic quantities (permittivity, permeability and electrical conductivity), the material parameters are typically strongly frequency dependent. As a result, the parameter h(ω) (and correspondingly $s_{2}={\left(h-1\right)}^{-1}$) can vary over several orders of magnitudes across a broad frequency range. This, in general, makes it possible to recover the SDF from experimental data, provided that measurements of the effective property are available over a sufficiently wide and densely sampled range of contrast parameter values, usually achieved by a wide frequency sweep.

Conversely, thermal conductivity, hydraulic conductivity and diffusion coefficient generally do not exhibit significant frequency dependence. Instead, these quantities may vary with temperature. The corresponding effective properties can be regarded as temperature-dependent, so that the contrast parameter can be tuned through temperature variation. A similar approach may be also applicable to magnetic properties in materials, where the permeability changes substantially with temperature, particularly near magnetic phase transition.

The SDF in Equation (3) is normalized to unity:

(4) $$\int_{0}^{1}g_{2}\left(x\right)dx=1-C_{2}.$$

In addition, for a system possessing a cubic or isotropic rotational symmetry, its first moment is known to satisfy the sum rule

(5) $$\int_{0}^{1}xg_{2}\left(x\right)dx=\frac{f_{1}}{d},$$

where *d* is the spatial dimensionality of the system.

The spectral density function for the classical Maxwell Garnett and Bruggeman theories is well known [33]. For the LE (1), this function was derived earlier in [34]. Although these result are correct, the SDF can be represented in a very neat compact form using complex notation. As can be checked, if k≠0, it is simply

(6) $$g_{2}\left(x\right)=\frac{1}{\pi f}ℑ{\left[1-f+ft^{k}\exp\left(ik\pi \right)\right]}^{1/k},$$

where t=1/x−1. For some particular cases, SDF takes especially simple forms. At k=1/2 (the so-called CRIM— complex-refractive-index-model) it is [26]

(7) $$g_{2}\left(x\right)=\frac{2}{\pi }\left(1-f\right){\left(\frac{1}{x}-1\right)}^{1/2}.$$

At k=1/3, for the so-called Landau-Lifshitz-Looyenga (LLL) equation [35,36]

(8) $$g_{2}\left(x\right)=\frac{3^{3/2}}{2\pi }\left(1-f\right){\left(\frac{1}{x}-1\right)}^{1/3}.$$

At k=0, for the LLE [34],

(9) $$g_{2}\left(x\right)=\frac{\sin\left(\pi f\right)}{\pi f}{\left(\frac{1}{x}-1\right)}^{f}.$$

Consider next the first moment of the SDF. Substituting Equation (6) into Equation (5) allows one to find a relationship between the parameters *k* and *d*. After bulky calculations, the final result is unexpectedly simple:

(10) $$d=2\frac{1+\frac{1}{6}k}{1-\frac{5}{6}k-\frac{1}{6}k^{2}}.$$

**Figure 1:**
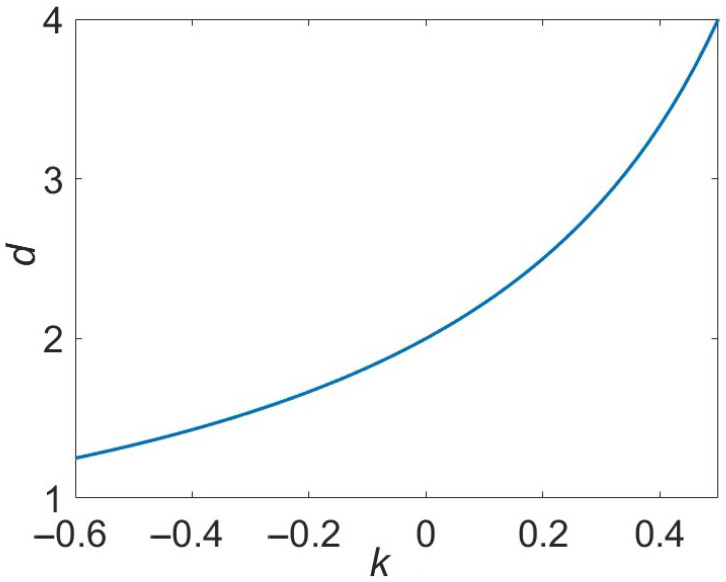
The dependence of d(k) in accordance with Equation (10).

The dependence d(k) is shown in Figure 1. In particular, we have d=2 at k=0, d=3 at k=1/3, and d=4 at k=1/2. As k→−1,d→1. Consider then the relationship between *k* and *d* in more details.

## 4. The Parameter *k* and the Issue of Dimensionality

In the case of k=−1, d=1 and the corresponding microgeometry can be imagined as a layered periodic system in which the displacement field, magnetic induction, electric or thermal currents, or heat or mass flux are directed normal to the layers. We note that this direction is the only one that is defined in one dimension. As the ratio of the 1D lattice period to a macroscopic length scale (such as the wavelength of light, characteristic scales of potential or temperature variation, or diffusion length) tends to zero, LE is known to be exact.

The case of k=0 yields d=2 and results in the LLE, Equation (2), which is particularly frequently used to describe various effective physical characteristics of composite media. The main results for 2D systems have been obtained with the use of the Keller-Dykhne duality relation [37,38].

It is well-known that in 2D systems with $f_{1}=f_{2}=1/2$, which possess phase-inversion symmetry, the equation

(11) $${\tilde{\epsilon }}_{eff}=\sqrt{\epsilon_{1}\epsilon_{2}}$$

holds, independently of the specific microgeometry. It is easy to see that Equation (11) follows from Equation (2) when $f_{1}=f_{2}=1/2$. In addition, using Equation (11) one can verify that Equation (2) coincides with the 2D symmetric Bruggeman equation.

What happens if $f_{1}\neq f_{2}$? Significant progress in this direction was achieved by Machavariani and Fel [39,40]. They began with the microgeometry of a 2D regular infinite checkerboard and assumed that each square on this checkerboard is itself non-uniform, being composed of smaller (finite) checkerboards. By repeating this recursive procedure indefinitely, Equation (2) can be recovered as the result of an interpolation. However, if the recursion is terminated at a finite stage, Equation (2) becomes approximate. It is important that due to the Keller-Dykhne duality, not only square lattices but also trianglular ones and random spot distributions lead to similar conclusions.

Naturally, the key point is the accuracy of this approximation. To quantify it, Machavariani performed numerical calculations of the effective conductivity for various regular 2D structures [40]. The obtained results show that the discrepancy between the numerically obtained effective conductivity and Equation (2) depends markedly on the phase contrast $\sigma_{1}/\sigma_{2}$, with the deviations remaining small for a low contrast, specifically for $0.2\leq \sigma_{1}/\sigma_{2}\leq 5$. These findings were shown to hold not only for two-phase composites but also for multi-phase systems.

Another interesting feature of the LLE was noted by Simpkin [3]. He found that under the condition of small Clausius-Mossotti factors,

(12) $$\left|\frac{\epsilon_{1}-\epsilon_{eff}}{\epsilon_{1}+2\epsilon_{eff}}\right|\ll 1$$

and

(13) $$\left|\frac{\epsilon_{2}-\epsilon_{eff}}{\epsilon_{2}+2\epsilon_{eff}}\right|\ll 1,$$

the symmetric 3D Bruggeman equation follows from Equation (2). It is obvious that the conditions (12) and (13) are satisfied under a low contrast assumption, i.e., when $0.5\ll |\epsilon_{2}/\epsilon_{1}|\ll 2$.

Of particular interest is the case of k=1/3, which corresponds to d=3 and yields the LLL equation,

(14) $$\epsilon_{eff}^{1/3}=f_{1}\epsilon_{1}^{1/3}+f_{2}\epsilon_{2}^{1/3}.$$

This equation was considered in detail by Dube [41] and Banhegui [42]. After analysing experimental data on the effective permittivity of dielectric powders at radio- and micro-wave frequencies, Dube concluded that Equation (14) is accurate (within 3%) up to the dielectric constant about 13, provided that the particle size exceeds 50 μm. At the same time, a comparison of experimental and calculated results for the loss factor shows much larger discrepancy, within 10–20%.

Banhegyi reviewed experimental data on the permittivity of near-spherical dielectric particles dispersed in various liquids for a wide range of filling fractions and pointed out that the LLL equation provides the best fit to experiments (within 1%) compared with several other EMA, except in the case of mineral particles in water. The latter case, however, is characterized by a relatively high dielectric contrast ($\epsilon_{1}/\epsilon_{2}\approx 13.4$), whereas the validity of the LLL equation is presumably limited to the regime $|\epsilon_{1}-\epsilon_{2}|\ll \epsilon_{eff}$, which requires a low (close-to-unity) value of $\epsilon_{1}/\epsilon_{2}$. Naturally, none of the above mixtures possesses phase-inversion symmetry. Overall, the author concluded that Equation (14) is not applicable to mixtures of nonspherical particles or to systems with high dielectric contrast, especially metal-dielectric composites.

The inconsistency of the LLL equation at high contrast *h* becomes evident when considering the simplest deterministic geometry possessing phase-inversion symmetry, such as a 3D regular checkerboard. In terms of conductivity, Equation (14) yields $\sigma_{eff}/\sigma_{1}\to \frac{1}{8}\sigma_{2}/\sigma_{1}$ as $\sigma_{2}\to \infty$, whereas the exact asymptotic result is known to be $\sigma_{eff}/\sigma_{1}\to 2\sqrt{\sigma_{2}/\sigma_{1}}$ [43].

Several of recent studies also demonstrate a good predictive capability of the LLL equation, in particular, for such composite systems as iron-based powders [44], fly ash [45], and mixed magnetite-maghemite particle composites [46].

## 5. Archie’s Law

If k<1/3, except for the special cases k=−1 and k=0, Equation (10) yields non-integer values of *d*. In addition, if k>1/3, then d>3. Since such values of *d* appear non-physical, this issue requires closer examination.

It is well known that for a wide variety of porous rocks with an insulating matrix saturated with a saline (conducting) fluid, the effective conductivity follows so-called Archie’s law [47]

(15) $$\sigma_{eff}=\sigma_{2}f_{2}^{m},$$

where $\sigma_{2}$ and $f_{2}$ are the conductivity and volume fraction (porosity) of the conducting fluid, respectively. The empirical parameter *m* is usually referred to as the cementation exponent. It is easy to see that Equation (15) follows directly from Equation (1)—expressed in terms of conductivity—under the high-contrast condition $\sigma_{1}/\sigma_{2}\ll 1$, with m=1/k [18]. Typically, values of m=1.5−2.5 (k=0.4−0.66) are characteristic of most porous arenaceous sediments, while values of m=2.5−5 (k=0.2−0.4) occur in some carbonates [47,48].

For our purposes, it is more convenient to consider not Equation (15), but its modified version, which assumes that $\sigma_{1}\neq 0$, so that both phases contribute to the effective conductivity. Modified Archie’s law is given by [49]

(16) $$\sigma_{eff}=\sigma_{1}{\left(1-f_{2}\right)}^{p}+\sigma_{2}f_{2}^{m},$$

where *m* and *p* are empirical constants. Imposing the obvious condition that if $\sigma_{1}=\sigma_{2}$, then $\sigma_{eff}=\sigma_{1}=\sigma_{2}$, one has ${\left(1-f_{2}\right)}^{p}+f_{2}^{m}=1$. This, in turn, allows Equation (16) to be rewritten as

(17) $$\sigma_{eff}/\sigma_{1}=1+f_{2}^{m}\left(\frac{\sigma_{2}}{\sigma_{1}}-1\right).$$

Comparing this with Equations (3) and (4) yields the only solution for the SDF: g(x)=0 and $C_{2}=f^{m-1}$. If this case, the second sum rule, Equation (5), breaks down, resulting in a zero first moment. Thus, strictly speaking, Archie’s law is hardly consistent with the spectral representation for isotropic media.

At first sight, the above inconsistency may seem surprising and contradictory. In fact, it is important to note that although Equation (15) follows from Equation (1), it is not vice versa. While the condition of topological percolation for both phases is typically satisfied for porous media obeying Archie’s law, the condition of phase-inversion symmetry is generally not met.

In Equation (5), the factor $d^{-1}$ comes from an angular average over the directions of the applied electric field [50]. If microgeometry is anisotropic, the first moment becomes tensorial (it contains a directional dependence). Furthermore, in some cases the spatial dependence *d* may become ill-defined. For instance, a quasi-2D microstructure is highly nonuniform (structured) within a plane (two directions) and nearly uniform or weakly varying along the third direction; a quasi-3D microstructure (almost isotropic 3D) may be approximately uniform along two directions and slightly non-uniform along the third. Moreover, such phenomenon as 2D-3D (or 3D-2D) crossover can occur [51,52,53,54].

## 6. The Analysis of Uncertainties

Because the values of the phase conductivities (permittivities) $\sigma_{i}$ ($\epsilon_{i}$) and their volume fractions $f_{i}$ are always known with some uncertainty, the measured effective permittivity or conductivity is inherently uncertain. Likewise, an error in the parameter *k* introduces uncertainty in the calculated effective quantity. The inaccuracy of the effective conductivity $\Delta \sigma_{eff}$ for the LE can be estimated as

(18) $$\Delta \sigma_{eff}=\frac{\partial \sigma_{eff}}{\partial k}\Delta k+\frac{\partial \sigma_{eff}}{\partial f}\Delta f+\frac{\partial \sigma_{eff}}{\partial \sigma_{2}}\Delta \sigma_{2}+\frac{\partial \sigma_{eff}}{\partial \sigma_{1}}\Delta \sigma_{1}.$$

Let us now evaluate the partial derivatives $I_{1}=\partial \sigma_{eff}/\partial k$, $I_{2}=\partial \sigma_{eff}/\partial f$ and $I_{3}=\partial \sigma_{eff}/\partial \sigma_{2}$ (the derivative $\partial \sigma_{eff}/\partial \sigma_{1}$ can be determined analogously to $I_{3}$ due to symmetry). After straightforward algebra, one obtains for k≠0:

(19) $$I_{1}^{\left(k\right)}=\frac{\sigma_{eff}}{k}\left[\frac{\left(1-f\right)\sigma_{1}^{k}\ln\sigma_{1}+f\sigma_{2}^{k}\ln\sigma_{2}}{\sigma_{eff}^{k}}-\ln\sigma_{eff}\right],$$

(20) $$I_{2}^{\left(k\right)}=\frac{\sigma_{eff}^{1-k}}{k}\left(\sigma_{2}^{k}-\sigma_{1}^{k}\right),$$

(21) $$I_{3}^{\left(k\right)}=f{\left(\frac{\sigma_{eff}}{\sigma_{2}}\right)}^{1-k}.$$

At k→0, using l’Hôpital’s rule, one has

(22) $$I_{1}^{\left(0\right)}=\frac{1}{2}f\left(1-f\right)\sigma_{eff}\ln^{2}\left(\sigma_{2}/\sigma_{1}\right),$$

(23) $$I_{2}^{\left(0\right)}=\sigma_{eff}\ln\left(\sigma_{2}/\sigma_{1}\right),$$

(24) $$I_{3}^{\left(0\right)}=f\sigma_{eff}/\sigma_{2}.$$

The values of the parameter *k* are usually obtained by fitting to experimental data. Since $f_{i}$ and $\sigma_{1}$ (or $\epsilon_{i}$) are known only with some uncertainty, the inaccuracy of the parameter *k* can be estimated as

(25) $$\Delta k=I_{1}^{-1}\left(\Delta \sigma_{eff}-I_{2}\Delta f-I_{3}\Delta \sigma_{2}-I_{4}\Delta \sigma_{1}\right).$$

It is therefore useful to estimate the partial derivative $I_{1}$.

**Figure 2:**
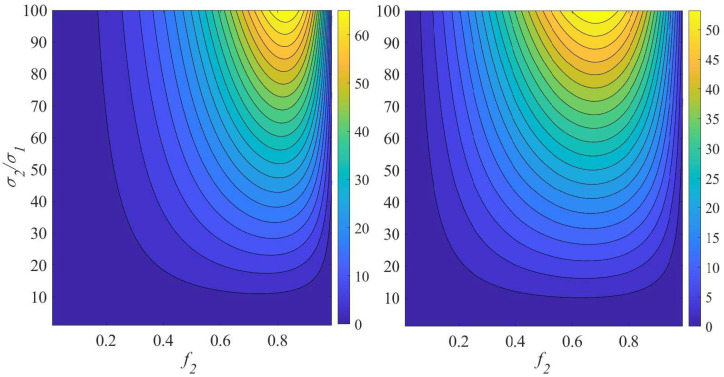
The partial derivatives $I_{1}^{\left(0\right)}$ (**left panel**) and $I_{1}^{\left(1/3\right)}$ (**right panel**).

If we are interested in small Δk, large values of the partial derivative $I_{1}$ are preferable. In the limit of $\sigma_{2}/\sigma_{1}\to 1$, both partial derivatives $I_{1}^{0}$ and $I_{1}^{\left(1/3\right)}$ are small and attain their maximum values at $f_{2}=1/2$. As seen in Figure 2, when the contrast $\sigma_{2}/\sigma_{1}$ increases, the peak of $I_{1}$ shifts monotonically from lower to higher values of $f_{2}$. In particular, at $\sigma_{2}/\sigma_{1}=100$, $I_{1}^{\left(1/3\right)}$ reaches its maximum value at $f_{2}≃0.68$, whereas $I_{1}^{\left(0\right)}$ reaches its maximum at $f_{2}≃0.83$. In all cases, the derivative $I_{1}$ is small either when the contrast *h* is low (i.e., close to unity) or when $f_{2}$ is close to 0 or 1. In the limit $\sigma_{2}/\sigma_{1}\to \infty$, the partial derivative $I_{1}$ reaches its maximum under the condition $\partial I_{1}/\partial f_{2}=0$. One can verify that, in this limit, the maximum of $I_{1}^{\left(0\right)}$ occurs as $f_{2}\to 1$, while the maximum of $I_{1}^{\left(1/3\right)}$ occurs at $f_{2}=e^{-1/3}≃0.717$.

## 7. Conclusions

In this paper, we have assessed applicability of widely used general Lichtenecker equation for isotropic composite media. The key findings of our study can be summarized as follows:

(1) The Lichtenecker equation is compatible with the spectral representation only at k→0 (logarithmic Lichtenecker equation) in 2D and k=1/3 (Landau-Lifshitz-Looyenga equation) in 3D.
(2) In 3D, the Lichtenecker equation is applicable only for low contrast *h*, even for composites possessing phase-inversion symmetry.
(3) Although Archie’s is confirmed in many experiments for porous media, this does not, by itself, validate the Lichtenecker equation.
(4) If the empirical parameter *k* of the Lichtenecker equation is determined by fitting to experimental data, small errors in phase volume fractions and/or their conductivities and permittivities can lead to significant inaccuracies in *k*, especially when $f_{2}$ is close to 0 or 1, or when the contrast parameter *h* is low.

In conclusion, great care must be taken when using the Lichtenecker equation, particularly for the effective permittivity or conductivity of metal-dielectric mixtures. Although it sometimes yields reasonable results, in most cases its use is not physically justified.

## Data Availability

The original contributions presented in this study are included in the article. Further inquiries can be directed to the corresponding author.
